# The effect of patient experience with nurses and ward type on intention to recommend: Focusing on integrated nursing and caring service wards and general wards, 2020–2022

**DOI:** 10.1371/journal.pone.0342582

**Published:** 2026-02-19

**Authors:** Jinsun Kim, Seungju Kim

**Affiliations:** Department of Health System, College of Nursing, The Catholic University of Korea, Seoul, Republic of Korea; Universiti Sains Malaysia, MALAYSIA

## Abstract

**Objective:**

This study aimed to compared inpatients’ intention to recommend hospitals between integrated nursing and caring service wards (INCSW) and general wards (GW), and examined between patients’ experiences with nurses and recommendation intention.

**Methods:**

This study analyzed 943 inpatients (INCSW = 223, GW = 720) using the Korea Medical Service Experience Survey (2020–2022). Intention to recommend and nurse experience were measured on 5-point Likert scales and treated as approximately continuous, a common approach that supports the use of multivariable linear regression. Ward differences were assessed using t-tests, and effect sizes were summarized using Cohen’s d.

**Results:**

Patients in INCSW reported higher recommendation intention than those in GW (Mean 4.11 vs 3.98), with a small effect size (Cohen’s d = 0.27). However, ward type was not independently associated with recommendation intention in the fully adjusted model. All nurse experience domains were positively associated with recommendation intention, with courtesy showing the largest coefficient (*β* = 0.27, 95% CI 0.17–0.37).

**Conclusion:**

Although recommendation intention was slightly higher among INCSW patients in unadjusted comparisons, ward type was not independently associated with willingness to recommend after adjustment. In contrast, all nurse experience domains were positively and significantly associated with patients’ willingness to recommend the hospital. Strengthening nurse communication competencies, supported by ward-level monitoring and feedback-based training, may enhance patient experience and willingness to recommend.

## Introduction

As healthcare systems shift from provider-centered to patient-centered models, patient experience has emerged as a key component in improving the quality of medical services [1,2]. Patients receive medical services from various healthcare providers at the hospital, and these experiences can influence their perceptions of the hospital and their utilization of medical services [2]. At the international level, the Organisation for Economic Co-operation and Development (OECD) has reported that 18 countries, including Australia, Canada, and the United Kingdom, systematically assess patient experience through the Patient-Reported Indicator Surveys [3]. In the UK, a national adult inpatient survey has been running since 2002 and is published annually [4]. In the United States, the Hospital Consumer Assessment of Healthcare Providers and Systems (HCAHPS), launched in 2006, has been widely used not only for public reporting and hospital evaluation but also for reducing readmission rates, strengthening patient-centered care, and providing financial incentives [5,6]. South Korea also launched a nationwide Medical Service Experience Survey (MSES) in 2017, which is conducted annually to collect and analyze patient experiences and utilize the findings for healthcare quality improvement and policy development [7]. Furthermore, patient experience survey results are disclosed to promote healthy competition among healthcare institutions and improve overall service quality [8].

Willingness to recommend a hospital reflects patients’ intentions to reuse services or recommend them to others [2,9–11] and is influenced by multiple factors, including physician-related factors, nursing care, hospital facilities, and administrative services. Negative experiences—such as unfriendly staff, long waiting times, or inconvenient facilities—can reduce patient satisfaction and the likelihood of recommendation [9,12–14]. In contrast, positive experiences contribute to higher satisfaction, improved clinical outcomes, and better perceived health status [1,2]. Such experiences often lead to loyalty behaviors, including return visits and continued service use, thereby supporting the sustainability and financial performance of healthcare organizations [10,11]. Because nurses have the most direct and continuous contact with patients, their interactions with patients are likely to play a central role in shaping recommendation intention [11,13,15].

In 2015, South Korea implemented the integrated nursing and caring service to improve the quality of nursing care and alleviate the caregiving burden on families [16,17]. Under this model, the government provides financial support through reimbursement to secure nursing staff, enabling hospitals to offer comprehensive inpatient care without the need for private caregivers [16–18]. Compared with general wards (GW), integrated nursing and caring service wards (INCSW) operate with lower nurse-to-patient ratios, allowing for more intensive nursing care [16,17,19,20]. Previous studies have reported that patients admitted to INCSW have lower unplanned readmission and higher satisfaction with nursing services than those in GW [21]. Furthermore, patients treated in INCSWs were more likely to recommend the hospital [22]. Patient experience assessments have consistently demonstrated that positive interactions with nurses significantly influence overall patient satisfaction and hospital recommendation [2,9,11,13,15]. Taken together, these findings suggest that both the characteristics of INCSWs and patients’ experiences with nurses contribute to their likelihood of recommending the hospital. However, studies that examine these factors concurrently remain limited, highlighting the need for further investigation.

Therefore, this study aimed to examine how ward type and patient experiences with nurses are associated with willingness to recommend a hospital, using data from MSES.

## Methods

This cross-sectional study used data from the MSES conducted from 2020 to 2022. The MSES is a nationwide survey conducted annually by the Ministry of Health and Welfare and the Korea Institute for Health and Social Affairs, targeting approximately 7,000 households across South Korea and including all household members aged 15 years and older. The survey includes health status, healthcare service utilization experiences, perceptions of the healthcare system, awareness of medical cost burdens, health check-up experiences, individual characteristics and household characteristics. As the MSES is publicly available and fully anonymized, no additional ethical approval or informed consent was required for this study. A total of 42,145 participants were included from 2020 to 2022. This study initially included 957 inpatients admitted to GW or INCSW. After excluding those under 20 years of age and participants with missing general characteristics, a total of 943 patients remained for analysis: 720 in GW and 223 in INCSW.

### Variables

The variables of interest are ward type and patient experience with nurses. The type of ward was assessed with one item on caregiving service experience, with responses categorized as “admitted to INCSW,” “employing a private caregiver,” and “not applicable.” Respondents selecting “admitted to INCSW” were classified as INCSW, and the remaining responses were classified as GW, consistent with the INCS model in Korea where personal or family caregivers are not allowed. The patient experience with nurses is measured by four factors in the question “What was the attitude of the nurse in charge you experienced at that time?” The evaluation tool for nursing service experience includes four items: courtesy of nurses, easy-to-understand explanations, responsiveness when needed, and discharge explanations. These were measured using a 5-point Likert scale. In this study, responses were analyzed from 1 point (“not at all true”) to 5 points (“very true”), with higher scores indicating a more positive nursing service experience.

The outcome variable is the intention to recommend the hospital. Participants were asked, “If someone in your vicinity intends to use this medical institution, would you recommend it to them?” This was assessed using one item from the healthcare utilization experience evaluation, measured on a 5-point Likert scale. In this study, responses ranged from 1 point (“not at all true”) to 5 points (“very true”), with higher scores indicating a more positive intention to recommend the healthcare institution.

The general characteristics of the participants included: sex(male, female), age (20–39, 40–59, 60 and older), education level (below primary, secondary, higher), income (Q1–Q5, Q1 has a low income level and Q5 has a high income level), year (2020 vs 2021–2022; early vs later pandemic period), and the presence of chronic diseases. The question regarding chronic diseases was: “Have you received treatment for any of the following chronic diseases in the past year? Chronic diseases include hypertension, diabetes, mental and behavioral disorders, respiratory diseases, heart diseases, cerebrovascular diseases, neurological disorders, cancer, thyroid disorders, liver diseases, chronic kidney failure, and others. If you have one or more chronic diseases, answer “yes”; if you do not have one, answer “no.”

### Statistical analysis

Descriptive comparisons between INCSWs and GWs were conducted using chi-square tests for categorical variables and t-tests for continuous variables. Associations between nurse experience domains and recommendation intention were examined using Pearson correlation analyses stratified by ward type. Recommendation intention, measured on a 5-point Likert scale, was treated as a continuous variable in multivariable linear regression models. Model diagnostics indicated no concerning multicollinearity (variance inflation factors [VIF] range: 1.05–3.63); residual summaries did not suggest major departures from normality (Shapiro–Wilk W = 0.98), and re-estimation using White heteroskedasticity-robust standard errors yielded consistent inferences. Sampling weights provided in the national survey were applied to all analyses; strata and cluster identifiers were not available. To assess misclassification from recoding “not applicable” as GW, we conducted sensitivity (excluding “not applicable”) and GW subgroup analyses (S1 Table). Differences in recommendation intention and nurse experience scores between wards were presented as weighted mean differences (INCSW − GW) with 95% confidence intervals (CIs) and the effect size Cohen’s d. In all analyses, a two-sided p-value < 0.05 was considered statistically significant. All statistical analyses were performed using SAS statistical software (version 9.4; SAS Institute Inc., Cary, NC, USA).

## Results

Table 1 shows the general characteristics of the study population by ward type. Among the 943 patients, 23.7% were admitted to INCSW and 76.4% to GW. Compared with GW patients, those in INCSW had a higher proportion of individuals aged ≥60 years, a lower proportion with higher education, and a higher proportion with chronic diseases. The distribution of survey year also differed by ward type, with GW admissions more frequent in 2020 and INCSW admissions more frequent in 2021–2022. In contrast, the distributions of sex and household income were broadly similar between the two ward types

**Table 1:** General characteristics of the study population.

Variable	Total(n = 943)		INCSW(n = 223)		GW(n = 720)		
	N	%	N	%	N	%	*p*
**Sex**							
Male	406	44.0	95	40.9	311	45.0	0.341
Female	537	56.0	128	59.1	409	55.0	
**Age**							
20–39	110	14.5	19	13.4	91	14.9	0.036
40–59	277	29.3	53	21.7	224	31.5	
60≤	556	56.2	151	64.9	405	53.6	
**Education**							
Below primary	216	20.1	51	21.1	165	19.8	0.005
Secondary	504	54.7	141	63.2	363	52.2	
Higher	223	25.2	31	15.8	192	27.9	
**Income**							
1Q	324	31.6	83	34.1	241	30.8	0.553
2Q	213	23.4	56	26.1	157	22.6	
3Q	151	16.3	35	16.1	116	16.3	
4Q	112	12.3	22	9.8	90	13.0	
5Q	143	16.4	27	13.9	116	17.2	
**Year**							
2020	494	54.1	78	40.9	416	58.0	<.0001
2021-2022	449	45.9	145	59.1	304	42.0	
**Chronic disease**							
Present	605	60.9	165	68.9	440	58.5	0.019
Absent	338	39.1	58	31.1	280	41.5	

All values are presented as *n* (survey-weighted %) using MSES person-level weights. *P* values are from survey-weighted chi-square tests.

Table 2 shows the differences in recommendation intention and nurse experience scores by ward type. Recommendation intention was higher in INCSW than in GW (Mean [M]: 4.11 vs. 3.98), with a mean difference of 0.13 (95% confidence interval [CI] 0.03–0.23, *p* = 0.012). The mean scores for all four nurse experience domains were also higher in INCSW than in GW, but the mean differences were small (0.01–0.08), and all *p*-values were ≥ 0.131. Among the four nurse experience domains, explanation had the highest mean score and response had the lowest mean score in both wards.

**Table 2:** Recommendation intention and patients’ experiences with nurses by ward type.

Variable	INCSW Mean	GW Mean	Mean differenceᵃ	S.E.	95% CIᵃ	Cohen’s d	*p*
**Recommendation intention**	4.11	3.98	0.13	0.05	0.03-0.23	0.27	0.012
**Experience with nurse’s services**							
Courtesy	4.29	4.22	0.07	0.04	−0.02-0.15	0.16	0.146
Explanation¹	4.35	4.27	0.08	0.05	−0.02-0.18	0.12	0.131
Response²	4.23	4.21	0.02	0.06	−0.10-0.14	0.00	0.743
Discharge³	4.27	4.26	0.01	0.05	−0.09-0.12	0.07	0.781

All estimates are survey-weighted using MSES person-level weights. Mean difference and 95% CI are calculated as INCSW minus GW. Cohen’s *d* indicates standardized mean differences.

¹Explanation = easy-to-understand explanation;

²Response = response when needed;

³Discharge = discharge explanation.

Table 3 shows the correlations between the four nurse experience domains and recommendation intention by ward type. In INCSW, the correlation with recommendation intention was highest for courtesy (*r* = 0.394), followed by discharge explanation (*r* = 0.339), explanation (*r* = 0.301), and response (*r* = 0.266) (all *p* < .0001). In GW, the correlations with recommendation intention were also highest for courtesy (*r* = 0.449), followed by explanation (*r* = 0.407), response (*r* = 0.372), and discharge explanation (*r* = 0.353) (all *p* < .0001). In both ward types, the four nurse experience domains were positively correlated with one another, and each domain was positively correlated with recommendation intention.

**Table 3:** Correlation between experience of nurse services and recommendation intention by ward type.

Variable	Courtesy	Explanation¹	Response²	Discharge³	Recommendation⁴
**INCSW**					
Courtesy	1.000				
Explanation	0.595 (p < .0001)	1.000			
Response	0.506 (p < .0001)	0.490 (p < .0001)	1.000		
Discharge	0.631 (p < .0001)	0.516 (p < .0001)	0.446 (p < .0001)	1.000	
Recommendation	0.394 (p < .0001)	0.301 (p < .0001)	0.266 (p < .0001)	0.339 (p < .0001)	1.000
**GW**					
Courtesy	1.000				
Explanation	0.588 (p < .0001)	1.000			
Response	0.548 (p < .0001)	0.477 (p < .0001)	1.000		
Discharge	0.516 (p < .0001)	0.485 (p < .0001)	0.468 (p < .0001)	1.000	
Recommendation	0.449 (p < .0001)	0.407 (p < .0001)	0.372 (p < .0001)	0.353 (p < .0001)	1.000

All values are survey-weighted Pearson correlation coefficients (*r*) using MSES person-level weights.

¹Explanation = easy-to-understand explanation;

²Response = response when needed;

³Discharge = discharge explanation;

⁴Recommendation = recommendation intention.

Table 4 shows the results of the multivariable linear regression analysis with recommendation intention as the outcome variable. The model adjusted for ward type, sex, age, educational level, household income, presence of chronic disease, survey year, and the four nurse experience domains. All four nurse experience scores were positively associated with recommendation intention, with regression coefficients of 0.27 for courtesy, 0.13 for explanation, 0.10 for response, and 0.09 for discharge explanation (all *p* < 0.05). Ward type was not significantly associated with recommendation intention (INCSW vs GW: *β* = 0.07, *p* = 0.133). Sensitivity and GW subgroup analyses addressing potential misclassification from ward-type recoding showed consistent positive directions for nurse-experience domains (S1 Table).

**Table 4:** Recommendation intention according to patients’ experiences with nurses.

Variable	*β*	S.E.	95% CI	P-value
**Ward**				
INCSW	0.07	0.05	−0.02-0.16	0.133
GW	Ref	–	–	
**Experience with nurse’s services**				
Courtesy	0.27	0.05	0.17-0.37	<.0001
Explanation¹	0.13	0.04	0.05-0.21	0.001
Response²	0.10	0.03	0.03-0.17	0.004
Discharge³	0.09	0.04	0.01-0.17	0.031
**Year**				
2021-2022	0.10	0.04	0.03-0.18	0.009
2020	Ref	–	–	
**Sex**				
Male	−0.03	0.04	−0.11-0.05	0.471
Female	Ref	–	–	
**Age**				
20–39	Ref	–	–	
40–59	−0.07	0.07	−0.21-0.07	0.345
60≤	−0.03	0.08	−0.18-0.12	0.703
**Education**				
Below primary	Ref	–	–	
Secondary	0.00	0.05	−0.10-0.10	0.997
Higher	0.01	0.08	−0.14-0.16	0.927
**Income**				
1Q	Ref	–	–	
2Q	−0.01	0.06	−0.12-0.11	0.922
3Q	0.00	0.06	−0.12-0.12	0.992
4Q	0.01	0.08	−0.13-0.16	0.861
5Q	−0.04	0.07	−0.17-0.10	0.578
**Chronic disease**				
Present	0.05	0.05	−0.05-0.14	0.338
Absent	Ref	–	–	

All estimates are from survey-weighted linear regression models using MSES person-level weights and are adjusted for ward type, survey year, sex, age group, educational level, household income quintile, presence of chronic disease, and the four nurse-experience domains. Reference categories were GW (ward), 2020 (year), female (sex), 20–39 (age), below primary education (education), 1Q (income), and no chronic disease.

¹Explanation = easy-to-understand explanation;

²Response = response when needed;

³Discharge = discharge explanation.

**Fig 1:**
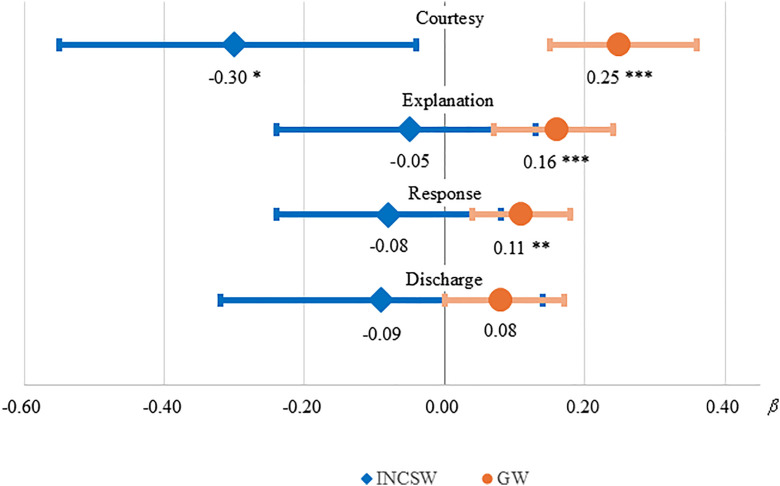
Associations between patient experience domains and intention to recommend by ward type. INCSW = integrated nursing and caring service ward; GW = general ward. Points indicate standardized regression coefficients (β), and horizontal lines indicate 95% confidence intervals. Estimates are from survey-weighted linear regression models adjusted for sex, age group, educational level, household income quartile, survey year, and presence of chronic disease. For visualization only, INCSW coefficients are displayed as −β to align directionality. * p < 0.05, ** p < 0.01, *** p < 0.001.

Fig 1 presents ward-stratified regression estimates for the associations between the four nurse-experience domains and recommendation intention. In both ward types, courtesy showed the largest coefficient. In INCSW, only courtesy was positively associated with recommendation intention (*β* = 0.30, 95% CI 0.04–0.55, *p* = 0.022), whereas the other domains had confidence intervals that included zero. In GW, courtesy (*β* = 0.25), easy-to-understand explanation (*β* = 0.16), and response when needed (*β* = 0.11) were positively associated with recommendation intention (all *p* < 0.01), while discharge explanation showed a weaker association that did not reach conventional significance (*p* = 0.064).

## Discussion

This study investigated the extent to which ward type and patients’ experiences with nurses are associated with willingness to recommend the hospital. Although INCSW patients showed higher recommendation intention in unadjusted comparisons, the independent association of ward type with recommendation intention was not pronounced after covariate adjustment. Conversely, patient experiences with nurses were positively associated with willingness to recommend, suggesting that patient-nurse interactions during hospitalization may have a stronger influence on willingness to recommend than ward type itself.

Although the association between INCSW use and willingness to recommend did not reach statistical significance in the present study, the direction of the findings was consistent with prior evidence [22]. This pattern may suggest that INCSW could influence not only quality-related aspects of inpatient care but also patient experience [1,22,23], which may translate into a greater willingness to recommend the hospital [2,9,11,12,22]. One plausible explanation is that INCSWs employ a skill-mix staffing model in which registered nurses work alongside nursing assistants and other nursing support staff, and they operate under a fixed nurse-to-patient ratio, resulting in relatively higher nurse staffing levels compared with general wards [16–20]. Such a configuration may facilitate more timely and comprehensive care and improve responsiveness to patient needs, thereby shaping patients’ perceptions positively [13,15,24–26]. Because this model in Korea is closely tied to a specific national policy context, generalizability to other health systems should be interpreted cautiously; however, the underlying principle of optimizing care delivery through an appropriate skill mix and adequate staffing may have broader international relevance. Nevertheless, given the lack of statistical significance, further research with larger and more diverse patient samples is warranted to more robustly evaluate the impact of integrated nursing care units on willingness to recommend.

In addition, this study suggests that nursing-related factors play an important role in patients’ willingness to recommend the hospital. All four nurse experience domains showed significant positive associations with recommendation intention, with the largest effect for courtesy, followed by easy-to-understand explanation, response when needed, and discharge explanation. This pattern is consistent with prior evidence that respectful attitudes, clear communication, and timely responses to patients’ needs are key determinants of patient satisfaction and recommendation intention [2,9,11–13,15]. Therefore, hospital and nursing managers should prioritize the ongoing management and support of frontline staff nurses who have the most frequent direct contact with patients, as their day-to-day interactions may shape patients’ overall perceptions of the hospital. Sustained workforce strategies—such as structured service and communication training, routine feedback mechanisms based on patient experience data, and unit-level monitoring coupled with coaching—may help maintain and improve patient-facing care quality and, ultimately, patients’ willingness to recommend.

Subgroup analyses indicated that the magnitude of the association between nurse experience and recommendation intention differed by ward type. While mean nurse-experience scores were higher in INCSW than in GW, the corresponding regression coefficients were relatively larger in GW, which may suggest ceiling effects in INCSW or greater responsiveness of recommendation intention to communication-related deficits (e.g., explanation and timely response) in GW. These ward-level differences may plausibly relate to variation in workload and the nursing work environment [24,27], which have been linked to patient experience [24] and to missed nursing care, including delays or omissions in patient-facing communication [15,24]. Accordingly, particularly in GWs, sustaining timely patient-facing communication may require workflow optimization together with nursing work environment–level supports, such as appropriate staffing allocation, workload adjustment, and, when needed, an appropriate skill mix [24]. However, because this study did not include objective ward- or shift-level staffing and structural indicators, these mechanisms could not be directly evaluated. Future studies should incorporate ward/shift-level measures (e.g., nurse-to-patient ratios, nursing hours per patient day, and skill mix) using longitudinal or multilevel designs to test pathways linking workload, communication quality, and recommendation intention.

This study has several strengths. It used nationwide MSES data, enhancing the generalizability of the findings. Recommendation intention showed a more pronounced independent association with nurse-related patient experience than with ward type. In addition, linking communication-competency training with ward-level monitoring may be useful for quality-improvement efforts [28–30].

However, several limitations should be noted. First, because of the cross-sectional design, causal inferences cannot be made regarding the associations of ward type or nurse experience with recommendation intention. Second, ward type was derived from a self-reported item, and misclassification is possible—particularly within the heterogeneous “not applicable” category—which may have attenuated the observed association for ward type; however, the overall interpretation was unchanged in sensitivity and GW subgroup analyses (S1 Table). Third, recommendation intention and nurse experience were measured using 5-point self-reported Likert scales, with mean scores exceeding 4.0 for some items, raising the possibility of ceiling effects and social desirability bias. Fourth, recommendation intention may be influenced by other factors, such as physician care and the hospital environment [9,13,14,31], which were not fully captured in this study. Finally, although sampling weights were applied to improve population representativeness, the dataset did not include strata or cluster identifiers. As a result, standard errors may not fully account for the complex sampling design, and inferences should be interpreted with caution. Future studies should incorporate objective ward- or shift-level staffing and structural indicators within longitudinal or multilevel designs and use post-pandemic data to test underlying mechanisms and assess the reproducibility of these findings

## Conclusion

This study found that patients’ experiences with nurses may be an important factor associated with willingness to recommend the hospital. These findings support strengthening nurse communication competencies through targeted training that emphasizes courtesy and communication clarity, and advancing quality-improvement efforts by linking feedback-based training to ward-level monitoring of these specific patient experience indicators. In parallel, staffing allocation and workload adjustment in GW may be needed to support consistent, timely patient-facing communication and to sustain improvements over time. Future studies should further examine causal pathways using longitudinal or multilevel designs that incorporate objective staffing measures and structural indicators.

## Supporting information

S1 Table Sensitivity and subgroup analyses for ward-type classification. (DOCX)
