# Supplementary material for: The effect of patient experience with nurses and ward type on intention to recommend: Focusing on integrated nursing and caring service wards and general wards, 2020–2022
Source: PLoS One. 2026 Feb 19;21(2):e0342582. doi: 10.1371/journal.pone.0342582 (PMC12919837; doi:10.1371/journal.pone.0342582)
Supplement: S1 Table — (DOCX) [file pone.0342582.s001.docx]

**Supporting information**

**S1 Table. Sensitivity and subgroup analyses for ward-type classification (survey-weighted regression).**

| Variable | Model 1 | | | Model 2 | | | Model 3 | | |
| --- | --- | --- | --- | --- | --- | --- | --- | --- | --- |
|  | *β* | 95% CI | *p* | *β* | 95% CI | *p* | *β* | 95% CI | *p* |
| Courtesy | 0.32 | 0.10 - 0.53 | 0.004 | 0.24 | 0.13–0.35 | <.001 | 0.26 | -0.19–0.71 | 0.248 |
| Explanation¹ | 0.05 | -0.07–0.18 | 0.403 | 0.18 | 0.08–0.27 | 0.001 | 0.07 | -0.12–0.25 | 0.493 |
| Response² | 0.05 | -0.09 - 0.19 | 0.487 | 0.12 | 0.05–0.20 | 0.001 | 0.09 | -0.20–0.38 | 0.534 |
| Discharge³ | 0.10 | -0.06 - 0.27 | 0.220 | 0.08 | -0.01–0.18 | 0.084 | 0.03 | -0.21–0.27 | 0.815 |

Note. All estimates are from survey-weighted linear regression models using MSES person-level weights and are adjusted for survey year, sex, age group, educational level, household income quintile, and presence of chronic disease. Reference categories were 2020 (year), female (sex), 20–39 (age), below primary education (education), 1Q (income), and no chronic disease. ¹Explanation = easy-to-understand explanation; ²Response = response when needed; ³Discharge = discharge explanation.

Model definitions.
Model 1 = excluding “not applicable” (comparison between INCSW and GW defined as “hired a personal caregiver” only): N = 296; R² = 0.204; Root MSE = 0.547.
Model 2 = GW subgroup (“not applicable”): N = 647; R² = 0.276; Root MSE = 0.544.
Model 3 = GW subgroup (“hired a personal caregiver”): N = 73; R² = 0.344; Root MSE = 0.477.
